# Long-Term Halocarbon Observations in an Urban Area of the YRD Region, China: Characteristic, Sources Apportionment and Health Risk Assessment

**DOI:** 10.3390/toxics12100738

**Published:** 2024-10-12

**Authors:** Yuchun Jiang, Anqi Zhang, Qiaoli Zou, Lu Zhang, Hanfei Zuo, Jinmei Ding, Zhanshan Wang, Zhigang Li, Lingling Jin, Da Xu, Xin Sun, Wenlong Zhao, Bingye Xu, Xiaoqian Li

**Affiliations:** 1 State Key Laboratory of Environmental Criteria and Risk Assessment, Chinese Research Academy of Environmental Sciences, Beijing 100012, China; 2 Zhejiang Ecological and Environmental Monitoring Center, Hangzhou 310012, China; 3 Zhejiang Key Laboratory of Ecological and Environmental Monitoring, Forewarning and Quality Control, Hangzhou 310012, China; 4 College of Environmental and Resource Sciences, Zhejiang Provincial Key Laboratory of Organic Pollution Process and Control, Zhejiang University, Hangzhou 310058, China

**Keywords:** halocarbons, long-term variations, the YRD region, potential source, health risk assessment

## Abstract

To observe the long-term variations in halocarbons in the Yangtze River Delta (YRD) region, this study analyzes halocarbon concentrations and composition characteristics in Shanxi from 2018 to 2020, exploring their origins and the health effects. The total concentration of halocarbons has shown an overall increasing trend, which is driven by both regulated substances (CFC-11 and CFC-113) and unregulated substances, such as dichloromethane, chloromethane and chloroform. The results of the study also reveal that dichloromethane (1.194 ± 1.003 to 1.424 ± 1.004 ppbv) and chloromethane (0.205 ± 0.185 to 0.666 ± 0.323 ppbv) are the predominant halocarbons in Shanxi, influenced by local and northwestern emissions. Next, this study identifies that neighboring cities in Zhejiang Province and other YRD areas are potentially affected by backward trajectory models. Notably, chloroform and 1,2-dichloroethane have consistently surpassed acceptable thresholds, indicating a significant carcinogenic risk associated with solvent usage. This research sheds light on the evolution of halocarbons in the YRD region, offering valuable data for the control and reduction in halocarbon emissions.

## 1. Introduction

Halocarbons are derivatives of hydrocarbons where one or more hydrogen atoms are replaced by halogen elements such as fluorine, chlorine, bromine, or iodine. They are generally denoted by the chemical formula RX, in which X represents the halogen element. The sources of halocarbons are complex, encompassing both natural sources such as biomass burning and marine emissions, as well as anthropogenic sources like refrigerants, fire extinguishing agents, coatings, packaging, biomedicine, and foam plastics [1,2,3,4,5,6].

Halocarbons are known to induce environmental impacts such as the depletion of the stratospheric ozone layer and the exacerbation of global warming phenomena [7,8,9,10]. In response, the international community has collaboratively established the Montreal Protocol to control the global emissions of halocarbons. Additionally, halocarbons exhibit inherent toxicity, manifesting as irritative effects on the integumentary system, pulmonary tissues, and respiratory tract mucosa [11,12,13,14,15]. Long-term exposure to halocarbons poses serious health risks to life and can cause irreversible damage. Since 2018, China has included dichloromethane, chloroform, trichloroethylene, and tetrachloroethylene in the national inventory of toxic and hazardous air pollutants [16]. Consequently, China released a prioritized control list for emerging pollutants, which contained halocarbons such as dichloromethane, chloroform, and hexachloro-1,3-butadiene.

As a global industrial hub, the concentration evolution of halocarbons in China has generated considerable scholarly attention. Previous studies have conducted long-term observations in the North China Plain (NCP) and the Pearl River Delta (PRD), clarifying the local concentration characteristics and changes [17,18,19,20,21,22]. Long-term observations at the background site in the NCP (observation period: 2003–2018) and the Greater PRD (observation period: 2001–2018) have both concluded that the concentrations of substances supposed to phase out under the Montreal Protocol after 2010 [23], such as CFC-11, CFC-113, CFC-12, and carbon tetrachloride, have decreased, while the concentrations of alternative substances continue to rise, such as HCFC-22 and HFC-134a used as a refrigerant [21,24].

However, researchers have also drawn the conclusion that non-regulated halocarbons such as dichloromethane and chloroform have surprisingly increased in these regions. Between 2020 and 2021, a comparison of dichloromethane in the Hong Kong region with the MLO (Mauna Loa Observatory) site revealed an increase of 347% [25]. In 2020, the concentration of dichloromethane in polluted air masses at the Shangdianzi station was 39% higher than that in clean air masses [26]. In 2016, the mixing ratio of chloroform in Beijing was 0.71 ppbv [27], but from 2018 to 2022, the concentration rose to the range of 0.89–1.5 ppbv [17].

The YRD region is recognized as a substantial source region of dichloromethane and chloroform. Studies have found that the emissions of dichloromethane and chloroform in the eastern part of China dominate global changes [28,29,30,31]. Claxton et al. calculated that the proportion of dichloromethane emissions in Asia increased from 68% to 89% between 2006 and 2017 [31], while An et al. pointed out that the eastern part of China, including the YRD region, is one of the main contributing areas [28]. Fang et al. focused on the emissions of chloroform in the eastern part of our country from 2007 to 2015 [29], and An et al. used domestic observation to estimate the national emissions of chloroform from 2010 to 2020 [30], both concluding that the emission trends and magnitudes of the eastern region of China are highly consistent with global emissions. In addition, there have been reports of substantial emissions of carbon tetrachloride into the atmosphere in the YRD region [32,33].

Recently, the number of halocarbon observations conducted in the YRD region has been limited, with observation periods typically not exceeding one year. This short duration is insufficient to capture the interannual variation characteristics of halocarbon concentrations. Studies by Fan and Li reported atmospheric concentrations of specific halocarbons in Nanjing in 2018 and Hangzhou in 2021, respectively, but did not provide a comprehensive analysis of halocarbons in general [34,35]. Li et al. investigated the variation in halocarbon concentrations in Hangzhou throughout the year 2021. However, their study lacked long-term observational data [36]. Furthermore, health risk assessments within the YRD region have mostly targeted different types of industrial parks [37,38]. There remains a need to investigate health risks and evolution patterns at integrated industrial and residential sites over extended time scales.

In this study, continuous observations were conducted from 2018 to 2022 at Shanxi in the YRD region. The researchers analyzed the pollution characteristics, traced the possible sources of pollution air masses and assessed the health effects of various halocarbons. The results are intended to guide future control measures by enhancing our understanding of the long-term variations and transport characteristics of halocarbons in the YRD region, as well as key toxic halocarbon species affecting the local population.

## 2. Materials and Methods

### 2.1. Study Area and Monitoring Methods

**Figure 1:**
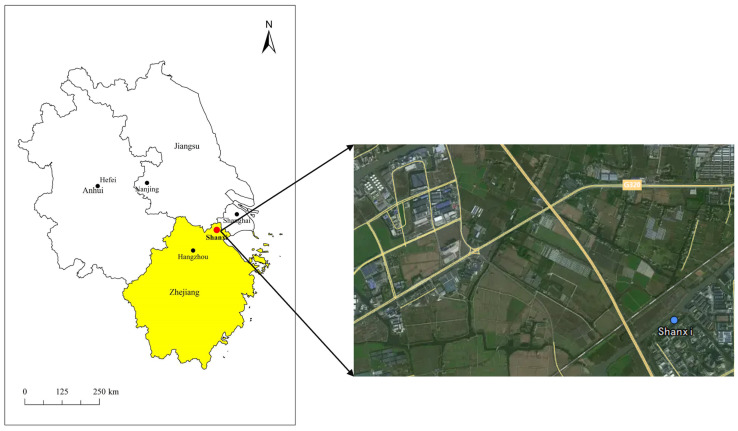
Geographic location of the Shanxi site.

Shanxi monitoring site (30°49′12″ N, 120°52′12″ E) is located in the central part of the YRD region, surrounded by important industrial cities of the region. To its northeast lies Shanghai, to the northwest is Nanjing, and to the southwest is Hangzhou. They are all capital cities in their provinces. Figure 1 illustrates the specific location of the observation site. The selection of the site can reflect the differences between regional transport and local emissions and then demonstrate the evolutionary characteristics of halocarbon in the YRD region. Furthermore, the site is situated in a mixed area of industrial and residential zones. The industry zones in the northwestern direction encompass general-purpose automotive machinery, electronic components manufacturing, and PVC production. Halocarbons have been largely used as solvents or raw materials in these industries [33,39,40,41]. There are also large areas of farmland and woodland in the south and north direction.

The study period spans from 1 January 2018 to 31 December 2022, during which 36,492 hourly data records were obtained, with a data validity rate of 83.3%.

In this study, the online monitoring of halocarbon concentrations was conducted using a Gas Chromatograph-Mass Spectrometer/Flame Ionization Detector system (TH-300B GC-MS, Tian Hong). Previous studies [42,43] have thoroughly described the operation of the GC-MS/FID system. In brief, the online GC-MS/FID system measures environmental halocarbons in three main steps: preparation, sampling and pre-concentration, and GC analysis. Pre-operation is conducted to minimize or prevent interference from previous samples. Additionally, the cold trap for impurity removal and enrichment trap for halocarbon capture are preheated to the respective temperatures for subsequent observations.

During the formal sampling phase, ambient air is passed through a cold trap that removes impurities such as O_3_, H_2_O, and CO_2_ and then channeled into a low-temperature cooling device, where it can be captured by a capillary column at ultra-low temperatures (−160 °C). Finally, the halocarbons are concentrated in the enrichment trap and are re-vaporized at 110 °C and injected into the GC-MS/FID system with a helium-carrier channel. During the analysis stage, a gas chromatograph, equipped with a DB-624 chromatographic column (30 m, ID 0.25 mm) and a mass spectrometer, is used to analyze the concentrations of halocarbons. Regular maintenance of the instrument was performed during the study period, including automatic zero-point calibration daily and multi-point calibrations monthly with concentrations of 0.4 ppbv, 0.8 ppbv, 1.2 ppbv, 2 ppbv, and 4 ppbv to ensure data accuracy.

### 2.2. Backward Trajectories and Bivariate Polar Coordinate Plots Model

The TrajStat model is essential for calculating backward trajectories, which helps in uncovering regional atmospheric transport patterns [44]. The meteorological data for the model are derived from the Air Resources Laboratory (ARL) (www.ready.noaa.gov/archive.php, accessed on 18 August 2024) of the National Oceanic and Atmospheric Administration (NOAA), with a grid resolution of 1° × 1° and hourly temporal resolution. Over the entire observational period, the configuration of the model is set to 3 days behind at the height of 100 m with a 1 h time interval (24 times a day, from 0:00 to 23:00 [local time]) [45,46]. It is designed to trace the possible sources of air masses reaching near the surface layer (100 m) [47]. The Euclidean distance clustering algorithm in TrajStat is employed for clustering air mass trajectories, facilitating the analysis of different types of transport air masses [48].

The bivariate polar plots model is able to allocate pollutant concentrations into sectors defined by ground wind direction and speed, thereby ascertaining the relationship between different pollutants and wind direction and speed. For a comprehensive introduction to this method, refer to the related work [49]. This technique involves averaging the data into various wind speed and direction intervals, followed by the application of the Kriging method for data interpolation, which leads to the generation of Bivariate polar plots.

**Figure 2:**
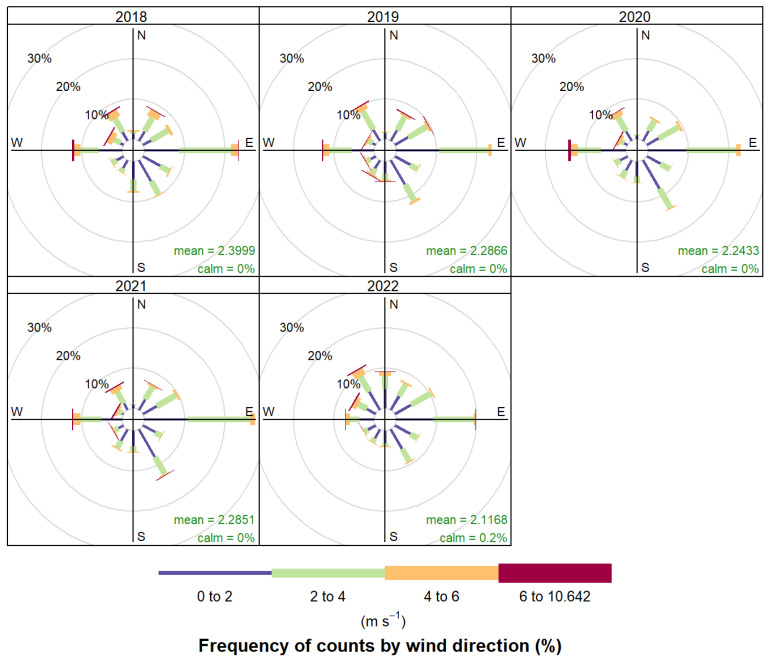
Wind rose diagrams illustrating the distribution of wind direction and frequency for the period 2018-2022 at the Shanxi site.

The meteorological parameters (wind speed, wind direction, temperature, and humidity) utilized in this study were all measured by an automatic weather station (WS500-UMB, Swarco-lufft, Germany). The general meteorological parameters at the Shanxi site from 2018 to 2022 are presented in Table S1, while the frequency distribution of wind speed and direction during the study period is depicted in Figure 2. As illustrated in the figure, the Shanxi site predominantly experiences east, west, and northwest winds, with wind speeds mostly ranging from 0 to 4 m/s. The methods can trace the spatial distribution and potential sources of pollutants at both regional and larger scales, and they have been extensively applied in the exploration of pollution source areas [50,51,52].

### 2.3. Environmental Health Risk Assessment

Human exposure to toxic and hazardous pollutants in the environment occurs through three primary pathways: inhalation, ingestion, and dermal absorption. Studies have shown that volatile halocarbons, due to their relatively high vapor pressures, primarily pose a risk through inhalation, making it the most significant route of exposure [53,54]. Consequently, to clarify the health risks of volatile halocarbons in ambient air, this study employs the health risk assessment methods recommended by the United States Environmental Protection Agency (US EPA, 2013) to evaluate the carcinogenic and non-carcinogenic risks associated with the inhalation way of volatile halocarbons within the study area. The formula for calculating the carcinogenic risk based on exposure concentration is as follows:

(1) R=IUR×EC

(2) EC=(CA×ET×EF×ED)/AT

In Equation (1), *R* represents the carcinogenic risk, *IUR* is the inhalation unit risk (μg/m^3^), *EC* is the exposure concentration (μg/m^3^), *CA* is the ambient air pollutant concentration (μg/m^3^), *ET* is the exposure time (hours per day), *EF* is the exposure frequency (days per year), *ED* is the exposure duration over a lifetime (years), and *AT* is the averaging time (hours).

The non-carcinogenic risk is assessed by the dimensionless hazard quotient (*HQ_i_*) for individual VOC species *i*. The HI denotes the non-cancer risk hazard of total halocarbons, which is calculated as follows:

(3) $$\begin{array}{l} HQ_{i}=EC_{i}/(RfC_{i}\times 1000)\\ HI=\sum_{i=1}^{n}HQ_{i} \end{array}$$

*RfC_i_* is defined as the concentration of a chemical substance, expressed in micrograms per cubic meter (μg/m^3^), which is considered safe for human inhalation over a lifetime without causing significant adverse health effects. *HQ_i_* is a dimensionless index used to assess non-cancer health risks. If the *HQ_i_* value exceeds 1, it indicates a potential for adverse non-cancer health effects. Conversely, an *HQ_i_* value of 1 or less suggests that adverse effects are unlikely.

In this study, *IUR* values for the halocarbons selected are obtained from the United States Environmental Protection Agency’s Integrated Risk Information System (IRIS) and the California Environmental Health Hazard Assessment Office (OEHHA); *RfC* values are sourced from IRIS. According to IRIS guidelines, *EF* is chosen as 365 days per year (d/y). Based on the Exposure Factors handbook for the Chinese adult population [55], *ET* varies by season and location, with 3.28 h per day (h/d) in spring and autumn, 3.17 h/d in summer, and 2.77 h/d in winter. *ED* and *AT* are set at 70.0 years and (70.0 × 365 × 24) hours, respectively. This study discusses the non-carcinogenic risks for several volatile halocarbons as well as the carcinogenic risks for others, with the *RfC* and *IUR* parameters for each species presented in Table S2.

## 3. Result and Discussion

### 3.1. Long-Term Characteristics of Halocarbons

In this study, we measured 29 halocarbons, including 19 alkyl halides, 8 alkenyl halides, and 5 aryl halides. The detailed information on measured species is shown in Table 1. The species in the table are ranked from high to low based on their contribution to the total concentration over the study time.

The proportion and the interannual variation of each chemical composition are shown in Figure S1. The statistical result indicates that the proportion of each component remained relatively stable over the years in Shanxi. Alkyl halides were the most abundant chemical composition, averaging 92.1% over a five-year period. In contrast, aryl halides and alkenyl halides fluctuated within the ranges of 3.5–7.8% and 0.9–3.1%, respectively (Figure S1).

We selected ten substances to reflect the general characteristics, which accounted for more than 90% of total halides. The major species, sequenced based on their overall contribution, are dichloromethane, chloromethane, 1,2-dichloroethane, Freon-11, carbon tetrachloride, 1,2-dichloropropane, trichloromethane, 1,1-dichloroethane, CFC-113, and trichloroethylene. 

**Figure 3:**
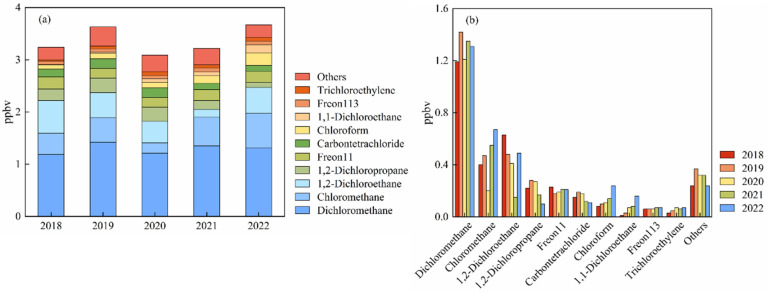
Time series of halocarbons at the Shanxi monitoring site from 2018 to 2022, showing (**a**) concentration levels and (**b**) chemical species composition.

Figure 3a,b illustrate the variations in levels and compositions of halon in Shanxi from 2018 to 2022. Aside from a notable decrease in 2020, the total concentration of halocarbons has shown an overall increasing trend. Specifically, the concentration increased by 19% from 2018 to 2019. However, there was a significant drop of 23.2% in 2020, with the total concentration falling from 3.605 ppbv to 2.767 ppbv. This decline is likely attributable to restrictions on industrial activities nationwide due to the abrupt onset of the COVID-19 pandemic [56,57]. The concentration rebounded in 2021 and 2022, reaching pre-pandemic levels of 3.215 ppbv and 3.676 ppbv, respectively.

As a substance phased out under the Montreal Protocol, CFC-11 in Shanxi decreased by 23% in 2019 but has since been gradually increasing, with an average annual growth rate of 6.6% from 2020 to 2022. Currently, the global CFC-11 concentrations have reaffirmed a downward trend, returning to pre-2013 levels by 2019 [58,59]. It has been reported that in Hangzhou, CFC-11 concentrations were 0.271 ppbv in 2016 [3] and 0.229 ppbv in 2021 [36], while in Nanjing, the concentration was 0.23 ppbv in 2018 [34]. These values suggest a decreasing trend in CFC-11 concentrations in other regions of the YRD region. A similar situation is observed with CFC-113. Despite the fact that the global concentration of CFC-113 is decreasing [4], CFC-113 fluctuated upward in this study, rising from 0.576 ppbv in 2018 to 0.069 ppbv in 2022. Although the concentration of CFC-11 in this study is slightly lower than that at background sites, the recent increase raises concerns about potential local CFC-11 and CFC-113 leaks considering the global downward trend. In contrast, carbon tetrachloride (CCl_4_) at Shanxi generally showed an obvious decline, consistent with global trend [4]. After peaking in 2019, carbon tetrachloride began to decrease, reaching an average of only 0.113 ppbv in 2022.

Dichloromethane and chloromethane are species that align with the overall trend of halocarbons, showing decline rates of 57% and 15%, respectively, in 2020. Dichloromethane returned to a pre-pandemic concentration level of 1.309 ppbv by 2022, whereas chloromethane showed an increase of 0.264 ppbv compared to that in 2018. 

This increase is attributed to industrial usage that releases substantial amounts of dichloromethane and chloromethane. Although natural sources are of importance for chloromethane, reports revealed that the enhancement in chloromethane in eastern China is primarily caused by anthropogenic sources [60], such as emissions from HCFCs/HFCs production and coal combustion [61]. Dichloromethane has been regarded as a typical tracer for various industries [62,63]. It is identified that the main sources of dichloromethane are the use of solvents, foam and plastic manufacturing, and emissions from HFC production [8,64]. The rapid increase in dichloromethane in ambient air has raised concerns. The results of this report indicate that a large fraction of the emissions originated from eastern China, including the YRD region [28].

Since 2018, 1,2-dichloroethane has experienced a year-by-year reduction in concentration, with an average decrease of 34.0% over four years. However, its concentration returned to 0.494 ppbv in 2022. This compound is a key raw material in the manufacture of vinyl chloride in the petrochemical industry as an intermediate compound using ethylene and Cl_2_ [39,65,66]. The “Jiaxing City Chemical Industry Development Plan” mentions that the growth rate of the local rubber and plastic products industry also slowed down from 2018 to 2020 [67].

In contrast, chloroform concentrations have continued to rise, unaffected by control measures in the pandemic. This persistence may be attributed to its emissions from the pharmaceutical manufacturing industry and its use as a solvent for extracting penicillin, antibiotics, or herbal plants [15,30,68]. According to public data from the Jiaxing Statistical Bureau, the output value of the pharmaceutical manufacturing industry in Jiashan County increased from 4.1267 billion yuan in 2018 to 5.3212 billion yuan in 2022 [69]. Additionally, the eastern region of Asia plays a significant role in chloroform emissions, contributing to the increase in global atmospheric concentrations of this substance from 2011 to 2017 [30]. Trichloroethylene also remains on the increase in concentration, primarily due to its use in dry cleaning and electronics manufacturing [46,68]. The YRD region has always been a crucial region of China’s electronics and pharmaceutical industries, and the changes in these substances are consistent with reality.

**Figure 4:**
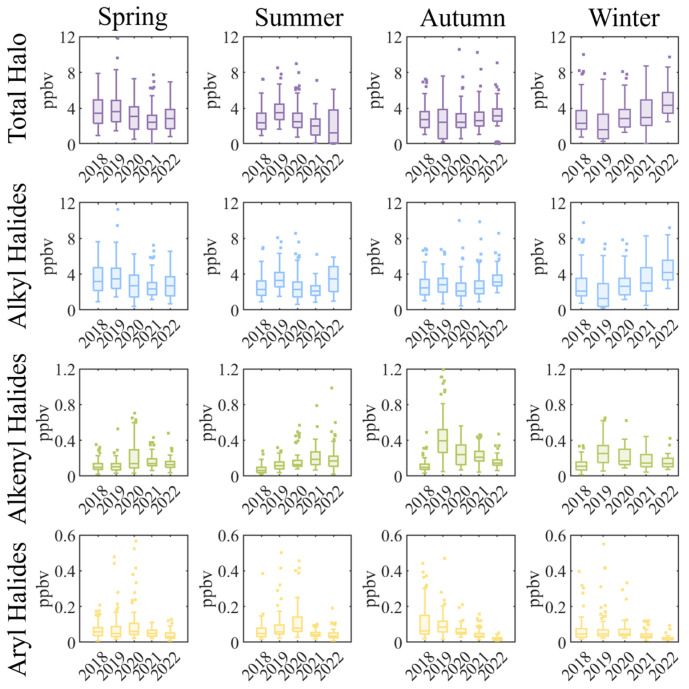
Seasonal variation in halocarbons at the Shanxi site.

To determine the seasonal characteristics of halocarbons, we defined spring as March to May, summer as June to August, autumn as September to November, and winter as December to February of the next year. Figure 4 illustrates the seasonal variations in halocarbons. At the Shanxi site, the total halocarbons ranged from 2.54–3.93 ppbv in spring, 1.96–3.71 ppbv in summer, 2.51–3.10 ppbv in autumn, and 2.32–4.75 ppbv in winter. Halocarbons show distinct seasonality, with higher concentrations in winter and lower levels in spring and summer. This may relate to stronger photochemical reactions and higher boundary layer heights in summer, which enhance the dilution of halocarbons. In contrast, stable atmospheric conditions in winter lead to the accumulation of these compounds, aligning with trends observed in previous studies [17,70].

Alkyl halides, due to their predominant presence, primarily drive the overall changes in total halocarbon concentrations. We observed that stringent control measures during the pandemic led to a slight decrease in halocarbon levels during the winter of 2019, as well as in the spring and summer of 2020. Following this period, alkyl halides showed an increase in concentration in subsequent years. Conversely, alkenyl halides and aryl halides exhibited a downward trend.

**Figure 5:**
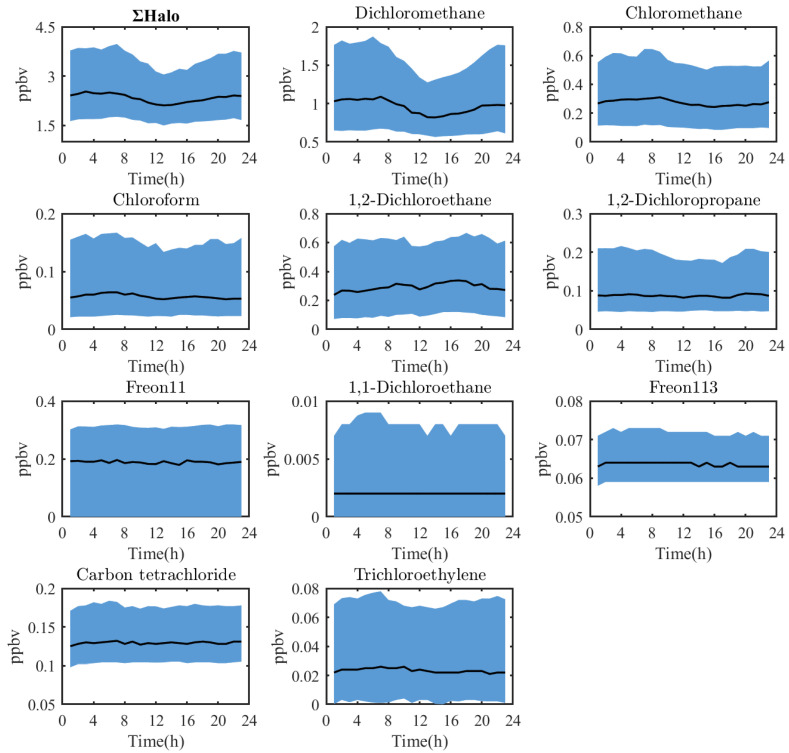
Diurnal variation of major halocarbon species and total halocarbons.

Figure 5 shows the diurnal variation of the main halocarbons. Due to extensive data gaps during the monitoring period, the variation of the 25th percentile for CFC-11 cannot be determined. The overall trend for total halocarbons followed a V-shaped pattern: concentrations began to decline from 7:00 to 8:00 (local time), reached their lowest point between 12:00 and 16:00 (local time), and then rebounded. Chloromethane, dichloromethane, and chloroform exhibited similar patterns. The daytime decrease is attributed to the elevated boundary layer and OH radical-dominated removal reactions, while the suppressed boundary layer height and the emitting sources facilitate accumulation during night and daytime, respectively [71]. The variability of halocarbon concentrations is also due to possible variations in the emitting sources, especially if the sources are close to the monitored area.

In contrast, 1,2-Dichloroethane showed a distinct pattern, with a new peak between 16:00 and 20:00 (local time), likely due to significant emissions from related factories. The third category, which includes Freon-11, carbon tetrachloride, 1,2-dichloropropane, CFC-113, 1,1-dichloroethane, and trichloroethylene. These substances have low reactive activity in the troposphere and, therefore, didn’t show significant concentration fluctuations.

To better understand the characteristics of halocarbons in the YRD region and the implementation of control measures, this study compiled sampling statistics of halocarbons in the NCP, the PRD, and the YRD regions, with the results presented in Table 2. Given the rapid industrialization and urbanization in China, only studies conducted within the same study period (2018–2022) were selected for comparison. Since the measured halocarbon species varied considerably, we simply compared the top ten species mentioned in this paper.

The concentration of chloroform in the Shanxi area is comparable to or slightly higher than that of other areas of the YRD. However, the annual average concentration of dichloromethane in Shanxi is lower than in other industrialized cities within the YRD region but higher than in cities of the NCP and the PRD. This can be ascribed to the different types of solvents used in different regions, with a higher utilization rate of dichloromethane solvents in the YRD region [34,36]. 

Additionally, the mixing ratios of 1,2-dichloroethane and trichloroethylene in the Shanxi and YRD regions are slightly higher. 1,2-Dichloroethane is mainly used in the production of vinyl chloride (PVC) [6,72]. Trichloroethylene is commonly used during degreasing processes in dry cleaning and in the metal or electronics manufacturing industries [12,46,73,74], indicating a thriving emerging electronics manufacturing and vinyl chloride manufacturing industry in the study area [18,71].

**Table 2:** Measured values of major halocarbons in the YRD, NCP, and PRD regions.

Region	Site	Year	CH_2_Cl_2_	CH_3_Cl	CH_2_ClCH_2_Cl	CFC-11	CCl_4_	CH_3_CHClCH_2_Cl	CHCl_3_	CFC-113	C_2_HCl_3_	Reference
YRD	Shanxi	2018	1.19	0.40	0.63	0.14	0.15	0.22	0.08	0.06	0.03	This study
	Shanxi	2019	1.33	0.47	0.47	0.16	0.18	0.28	0.09	0.07	0.05	This study
	Shanxi	2020	1.21	0.21	0.41	0.18	0.18	0.27	0.10	0.06	0.07	This study
	Shanxi	2021	1.20	0.51	0.15	0.19	0.11	0.17	0.06	0.07	0.07	This study
	Shanxi	2022	1.10	0.64	0.49	0.11	0.09	0.10	0.17	0.07	0.07	This study
	Nanjing	2018/07-2018/08	1.26	0.16	0.95	0.23	0.12	0.57	0.17	0.08	0.13	[34]
	Hangzhou	2021/01–2021/02	2.21	0.91	0.60	0.23	0.08	0.31	0.13	-	-	[35]
	Hangzhou	2021/01–2021/12	1.77	0.66	0.71	0.22	0.09	0.24	0.11	0.07	0.12	[36]
PRD	HongKong	2020/11–2021/06	0.36	0.88	-	0.24	0.09	-	0.06	0.08	0.05	[25]
	Guangzhou	2021/09–2021/10	0.99	0.09	0.15	-	-	0.11	-	0.09	0.07	[75]
NCP	Beijing	2018	0.89	0.55	0.49	0.37	0.12	0.19	0.36	0.07	0.02	[17]
	Beijing	2019	1.20	0.83	0.51	0.36	0.19	0.58	1.50	0.09	0.08	[17]
	Beijing	2020	1.10	0.49	0.53	0.84	0.18	0.15	0.29	0.07	0.05	[17]
	Taishan	2018/03–2018/04	0.39	0.85	-	0.25	0.09	-	0.18	0.08	0.01	[24]
	Shangdianzi (BG)	2020/10–2021/09	0.08	0.53	-	-	0.08	-	0.01	0.07	-	[26]
	Shangdianzi (Polluted)	2020/10–2021/09	0.47	0.81	-	-	0.08	-	0.06	0.07	-	[26]
	Handan	2018/05–2018/06	1.29	1.63	1.83	0.23	0.05	0.22	0.12	0.04	0.03	[18]

CH_2_Cl_2_: Dichloromethane; CH_3_Cl: Chloromethane; CH_2_ClCH_2_Cl: 1,2-Dichloroethane; CFC-11: Freon-11; CCl_4_: Carbon tetrachloride; CH_3_CHClCH_2_Cl: 1,2-Dichloropropane; CHCl_3_: Chloroform; CHCl_2_CH_3_: 1,1-Dichloroethane; CFC-113: Freon-113; C_2_HCl_3_: Trichloroethylene. The unit of these species is ppbv.

### 3.2. Main Potential Sources of Halocarbons 

**Figure 6:**
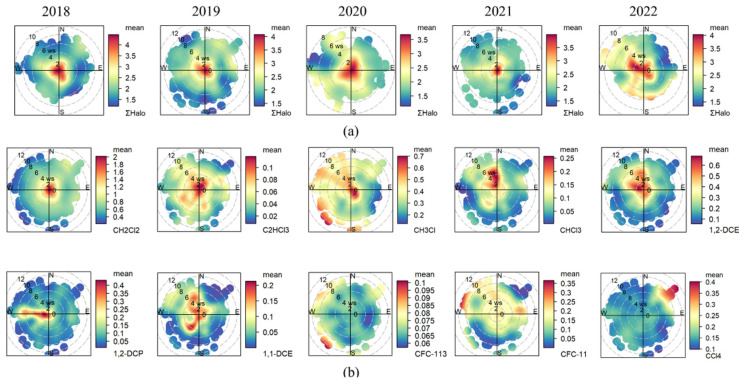
Main potential source regions of halocarbons. (**a**) represents the total halocarbons, and (**b**) focuses on a specific substance, as indicated in the lower right corner.

Bivariate polar plots were employed to investigate the impact of air mass transport from different regions on local halocarbons. The results are shown in Figure 6.

Figure 6a demonstrates the change in sources of total halocarbons over the years. From 2018 to 2021, local emissions were predominant, but with the resurgence of halocarbon in 2022, hotspots also appeared in the northwest. Different halocarbons exhibit variability in their potential geographical source areas. As shown in Figure 6b, dichloromethane and trichloroethylene primarily originate from emissions surrounding the monitoring site during the entire study. High-concentration areas are only associated with wind speeds of 0–2 m/s in all directions, indicating that dichloromethane is mainly generated by local emissions [76]. The nearby manufacturing sectors, including garments, pharmaceuticals, and electrical machinery [71], have a significant demand for the solvent dichloromethane. 

In addition to local emissions, trichloromethane, chloromethane, and 1,2-dichloroethane also have noticeable hotspots in the northwest wind direction. There are industrial activity areas such as electronic component processing plants [73,77,78] and vinyl chloride production [18,39,72] situated in that direction, which are possible sources of these substances. Chloromethane is also exhibited relatively high in the southwest, where extensive farmland is present, and is probably linked to biomass burning [79] and pesticide use [13]. 1,2-Dichloropropane showed a significant hotspot in the west, possibly due to automobile parts manufacturers [80]. In contrast, CFC-11, CFC-113, and CCl_4_ are evenly distributed across the study area. Occasionally, elevated levels of CFC-11 and CFC-113 align with industrial sources of halocarbons previously mentioned.

**Figure 7:**
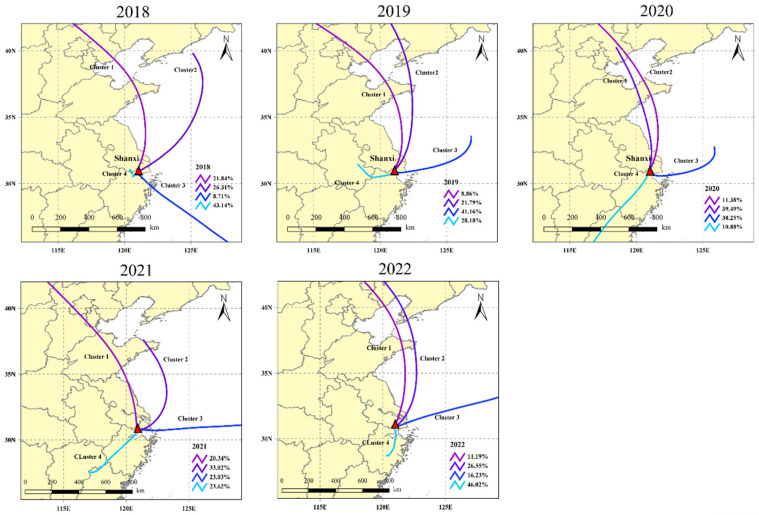
Possible sources of tracer halocarbons for air pollution transport during 2018–2019 at the Shanxi site.

This study also analyzed long-term (2018–2022) backward trajectories, a method previously used to study air mass transport in other areas of China. As shown in Figure 7, four distinct clusters were identified. Figure S2 illustrates the heights of these clusters. Cluster 1 and Cluster 2 represent northern trajectories, accounting for 14.72% and 29.43% of the total trajectories, respectively. However, Cluster 1, with an air pressure higher than 700 hPa and a longer transport range, had a lower likelihood of mixing with surface pollution compared to Cluster 2. In addition to local emissions, Cluster 3 is primarily influenced by marine air. Representing trajectories from the southwest direction, Cluster 3 is less affected by transport from anthropogenic emission sources and mainly consists of clean marine air. This cluster accounts for 31.84% of all back trajectories. Cluster 4, representing trajectories from the southeast direction and accounting for 30.37% of the total trajectories, was primarily influenced by local emissions from 2018 to 2019. However, in subsequent years, there was a noticeable shift towards emissions from other regions in Zhejiang Province, indicating an increase in the contribution of transport emissions from these areas.

### 3.3. Health Risk Assessment of Halocarbons

**Figure 8:**
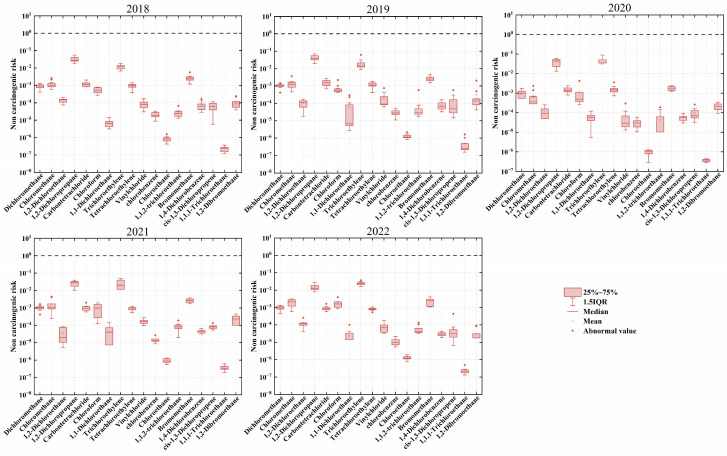
Interannual variation in non-carcinogenic risk of atmospheric halocarbons in Shanxi. The dashed line indicates the threshold value.

With the highest year being 2020, the average HI for the 18 halocarbons ranges from 0.046 to 0.077, implying that they posed no non-carcinogenic risk during the sampling time (Table S3). According to the US EPA standards, there are no halocarbons in the region that pose a non-cancer risk to humans (Figure 8). However, in the Shanxi region, trichloroethylene and 1,2-dichloropropane are substances with non-cancer risks close to the threshold from 2018 to 2022, with their five-year ranges being 0.013–0.035 and 0.014–0.044, respectively, both peaking in 2020. The non-cancer risk magnitudes for other substances like methyl bromide, dichloromethane, chloroform, and carbon tetrachloride remain around 10^−3^ with little variation. 1,2-Dichloropropane is an organic solvent mainly used in industrial coatings and inks [72], while trichloroethylene is a widely used degreaser in dry cleaning, metal, and automotive industries [73]. Similar conclusions have been found in risk assessments conducted in other cities in China, where 1,2-dichloropropane and trichloroethylene exhibit higher non-cancer risks among halocarbons, with some areas exceeding the EPA threshold [81,82]. Compared to these studies, the non-cancer risks at the Shanxi site are not significant, but there remains a high carcinogenic risk in the region.

**Figure 9:**
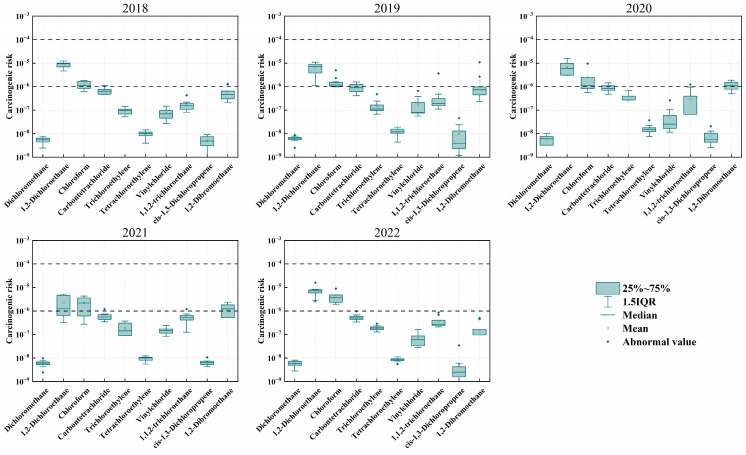
Interannual variation in carcinogenic risk of atmospheric halocarbons in Shanxi. The dashed lines indicate the acceptable risk level (1 × 10^−6^) and the tolerable risk level (1 × 10^−4^).

In Figure 9, the carcinogenic risks of chloroform and 1,2-dichloroethane consistently exceed the acceptable range throughout the monitoring period but remain within tolerable levels. Their ranges are 1.12 × 10^−6^ to 3.62 × 10^−6^ and 1.24 × 10^−6^ to 9.47 × 10^−6^, respectively. The carcinogenic risk of chloroform is continuously increasing, while the risk of 1,2-dichloroethane fluctuates significantly due to human activities. In contrast, the carcinogenic risks of carbon tetrachloride and 1,2-dibromoethane remain close to acceptable levels. The carcinogenic risk of carbon tetrachloride follows the overall concentration trend, rising and then falling, but still stays near acceptable levels. This may be due to the strong stability of carbon tetrachloride in the environment, allowing it to persist in the atmosphere for a long time and making its carcinogenic risk more noticeable over long periods than short-lived halocarbons. 1,2-Dibromoethane, however, exceeded the safety threshold for carcinogenic risk in 2020 and 2021. This substance is primarily used as a chemical intermediate in pesticides, dyes, resins, waxes, and adhesives and as a precursor to synthetic vinyl chloride [83]. The halocarbons that exceed acceptable carcinogenic risk levels in this study also show high carcinogenic risk in other cities [46,84].

The research findings imply that, despite the relatively low concentration proportions of certain halogenated hydrocarbons such as 1,2-dichloroethane, 1,2-dichloropropane, and trichloroethylene, they exhibit a higher level of carcinogenic and non-carcinogenic risks compared to the more concentrated dichloromethane and trichloromethane. These substances warrant particular attention in future regulatory control and emission reduction efforts.

## 4. Conclusions

Continuous observations on an hourly basis of atmospheric halocarbons are carried out in Shanxi, the central part of the YRD region, from 2018 to 2022. In this region, the pandemic disrupted the increase in halocarbons from 2018 to 2019, with concentrations only returning to previous levels by 2022. The most abundant halocarbons are dichloromethane and chloromethane, accounting for 52%. Notably, regulated substances such as CFC-11 and CFC-113 are on the rise. Additionally, the concentration of chloroform has been steadily increasing, which may be related to heightened emissions from the pharmaceutical industry under pandemic conditions. Furthermore, the potential source areas for halocarbons at the Shanxi site are local emissions from the cities within the YRD. Health risk assessments indicate that during the study period, no substances exceeded the non-carcinogenic risk threshold; however, trichloroethylene and 1,2-dichloropropane were close to the threshold. Notably, chloroform and 1,2-dichloroethane consistently exceeded the acceptable carcinogenic risk threshold throughout the study period. These substances may originate from solvents used in metal degreasing, automotive painting, pharmaceutical synthesis, and plastic manufacturing.

## Figures and Tables

**Table 1:** The mixing ratio and the contribution of species over the study period.

Unit ppbv	2018	2019	2020	2021	2022	%
Dichloromethane	1.194 ± 1.003	1.424 ± 1.004	1.214 ± 0.281	1.351 ± 1.241	1.309 ± 0.390	38.6%
Chloromethane	0.401 ± 4.003	0.475 ± 4.004	0.205 ± 0.185	0.548 ± 0.835	0.666 ± 0.323	13.4%
1,2-Dichloroethane	0.632 ± 6.003	0.478 ± 6.004	0.410 ± 0.171	0.151 ± 0.286	0.494 ± 0.255	12.8%
1,2-Dichloropropane	0.217 ± 2.003	0.278 ± 2.004	0.266 ± 0.087	0.172 ± 0.271	0.099 ± 0.041	6.2%
Freon11	0.228 ± 2.003	0.175 ± 2.004	0.188 ± 0.091	0.206 ± 0.672	0.212 ± 0.073	6.0%
Carbon tetrachloride	0.149 ± 1.003	0.185 ± 1.004	0.179 ± 0.054	0.118 ± 0.081	0.113 ± 0.033	4.4%
Chloroform	0.082 ± 0.003	0.098 ± 0.004	0.113 ± 0.030	0.142 ± 0.263	0.242 ± 0.138	4.0%
1,1-Dichloroethane	0.005 ± 0.003	0.034 ± 0.004	0.068 ± 0.002	0.076 ± 0.265	0.163 ± 0.126	2.0%
Freon113	0.058 ± 0.003	0.065 ± 0.004	0.063 ± 0.007	0.072 ± 0.020	0.069 ± 0.006	1.9%
Trichloroethylene	0.032 ± 0.003	0.054 ± 0.004	0.071 ± 0.010	0.065 ± 0.095	0.068 ± 0.015	1.7%
Tetrachloroethylene	0.043 ± 0.003	0.053 ± 0.004	0.067 ± 0.012	0.041 ± 0.039	0.037 ± 0.005	1.4%
Vinyl chloride	0.027 ± 0.003	0.063 ± 0.004	0.020 ± 0.015	0.052 ± 0.180	0.025 ± 0.015	1.1%
chlorobenzene	0.034 ± 0.003	0.051 ± 0.004	0.054 ± 0.013	0.024 ± 0.054	0.018 ± 0.008	1.1%
Chloroethane	0.026 ± 0.003	0.037 ± 0.004	0.029 ± 0.012	0.027 ± 0.049	0.039 ± 0.011	0.9%
1,1,2-trichloroethane	0.015 ± 0.003	0.034 ± 0.004	0.019 ± 0.008	0.049 ± 0.045	0.030 ± 0.017	0.9%
Bromomethane	0.026 ± 0.003	0.027 ± 0.004	0.018 ± 0.011	0.026 ± 0.038	0.022 ± 0.009	0.7%
cis-1,2-Dichloroethene	0.001 ± 0.003	0.027 ± 0.004	0.035 ± 0.001	0.023 ± 0.029	0.023 ± 0.032	0.7%
1,1-Dichloroethene	0.003 ± 0.003	0.023 ± 0.004	0.024 ± 0.001	0.018 ± 0.025	0.008 ± 0.011	0.5%
Freon114	0.010 ± 0.003	0.009 ± 0.004	0.013 ± 0.003	0.017 ± 0.011	0.013 ± 0.004	0.4%
1,4-Dichlorobenzene	0.015 ± 0.003	0.016 ± 0.004	0.012 ± 0.009	0.010 ± 0.006	0.007 ± 0.002	0.4%
1,2-Dichlorobenzene	0.009 ± 0.003	0.008 ± 0.004	0.010 ± 0.004	0.009 ± 0.009	0.005 ± 0.002	0.2%
Benzylchloride	0.016 ± 0.003	0.002 ± 0.004	0.004 ± 0.047	0.003 ± 0.004	0.001 ± 0.001	0.2%
1,3-Dichlorobenzene	0.006 ± 0.003	0.005 ± 0.004	0.005 ± 0.005	0.005 ± 0.011	0.002 ± 0.001	0.1%
cis-1,3-Dichloropropene	0.002 ± 0.003	0.004 ± 0.004	0.003 ± 0.001	0.003 ± 0.004	0.002 ± 0.004	0.1%
1,1,1-Trichloroethane	0.002 ± 0.003	0.003 ± 0.004	0.003 ± 0.001	0.003 ± 0.002	0.002 ± 0.001	0.1%
1,2-Dibromoethane	0.001 ± 0.003	0.003 ± 0.004	0.002 ± 0.001	0.002 ± 0.002	0.000 ± 0.000	0.0%
trans-1,3-Dichloropropene	0.001 ± 0.003	0.001 ± 0.004	0.001 ± 0.001	0.001 ± 0.002	0.002 ± 0.002	0.0%
Trans-1,2-Dichloroethene	0.000 ± 0.003	0.000 ± 0.004	0.000 ± 0.000	0.003 ± 0.010	0.003 ± 0.004	0.0%
Bromodichloromethane	0.001 ± 0.003	0.002 ± 0.004	0.000 ± 0.000	0.000 ± 0.000	0.001 ± 0.001	0.0%

Concentrations are represented as mean values ± standard deviation, with the last column depicting the average contribution of each species to the total concentration over the five-year period.

## Data Availability

The data are available on request from the corresponding author.

## Supplementary Materials

The following supporting information can be downloaded at https://www.mdpi.com/article/10.3390/toxics12100738/s1, Table S1: Meteorological parameters from 2018 to 2022. Table S2: RfC and IUR values for VOC species in this study. Table S3: Hazardous Index from 2018 to 2022. Figure S1: Proportion of halocarbon chemical components from 2018 to 2022. Figure S2: The height and contribution of each source during 2018–2022.
