# Supplementary material for: Long-Term Halocarbon Observations in an Urban Area of the YRD Region, China: Characteristic, Sources Apportionment and Health Risk Assessment
Source: Toxics. 2024 Oct 12;12(10):738. doi: 10.3390/toxics12100738 (PMC11511214; doi:10.3390/toxics12100738)
Supplement: Supplementary file 1 [file toxics-12-00738-s001.zip › toxics-3210775-supplementary.pdf]

Supplementary materials

Table S1 Meteorological parameters from 2018 to 2022.

Sampling period	Temperature (℃)	Relative humidity （%）	Relative pressure （hPa）	wind speed （m/s）
2018	17.42	80.04	1015.79	2.4
2019	17.63	79.78	1015.38	2.29
2020	18.13	79.99	1015.69	2.24
2021	18.41	78.39	1015.38	2.29
2022	17.80	79.13	1026.55	2.33

Table S2 RfC and IUR values for VOCs species in this study.

Species	Rfc (mg/m^3)	IUR ($m^3/\mu g$)	Species	Rfc (mg/m^3)	IUR ($m^3/\mu g$)
Chloromethane	9.00×10^{-2}	-	1,2-Dichloroethane	2.40×10^{0}	2.60×10^{-5}
Bromomethane	5.00×10^{-3}	-	1,2-Dichloropropane	4.00×10^{-3}	-
Chloroethane	1.00×10^{1}	-	cis-1,3-Dichloropropene	2.00×10^{-2}	4.00×10^{-6}
1,1-Dichloroethene	2.00×10^{-1}	-	1,1,2-trichloroethane	4.00×10^{-1}	1.60×10^{-5}
Dichloromethane	6.00×10^{-1}	1.00×10^{-8}	Tetrachloroethylene	4.00×10^{-2}	2.60×10^{-7}
Chloroform	9.80×10^{-2}	2.30×10^{-5}	1,4-Dichlorobenzene	8.00×10^{-1}	1.10×10^{-5}
Carbontetrachloride	1.00×10^{-1}	6.00×10^{-6}	1,1-Dichloroethane	5.00×10^{-1}	1.60×10^{-6}
1,2-Dibromoethane	9.00×10^{-3}	6.00×10^{-4}	1,2,4-Trichlorobenzene	2.00×10^{-1}	-
chlorobenzene	1.00×10^{0}	-	-	-	-

Table S3 Hazardous index from 2018 to 2022.

Sampling period	HI (average)	HI (median)
2018	0.052	0.047
2019	0.071	0.060
2020	0.077	0.085
2021	0.058	0.056
2022	0.049	0.046

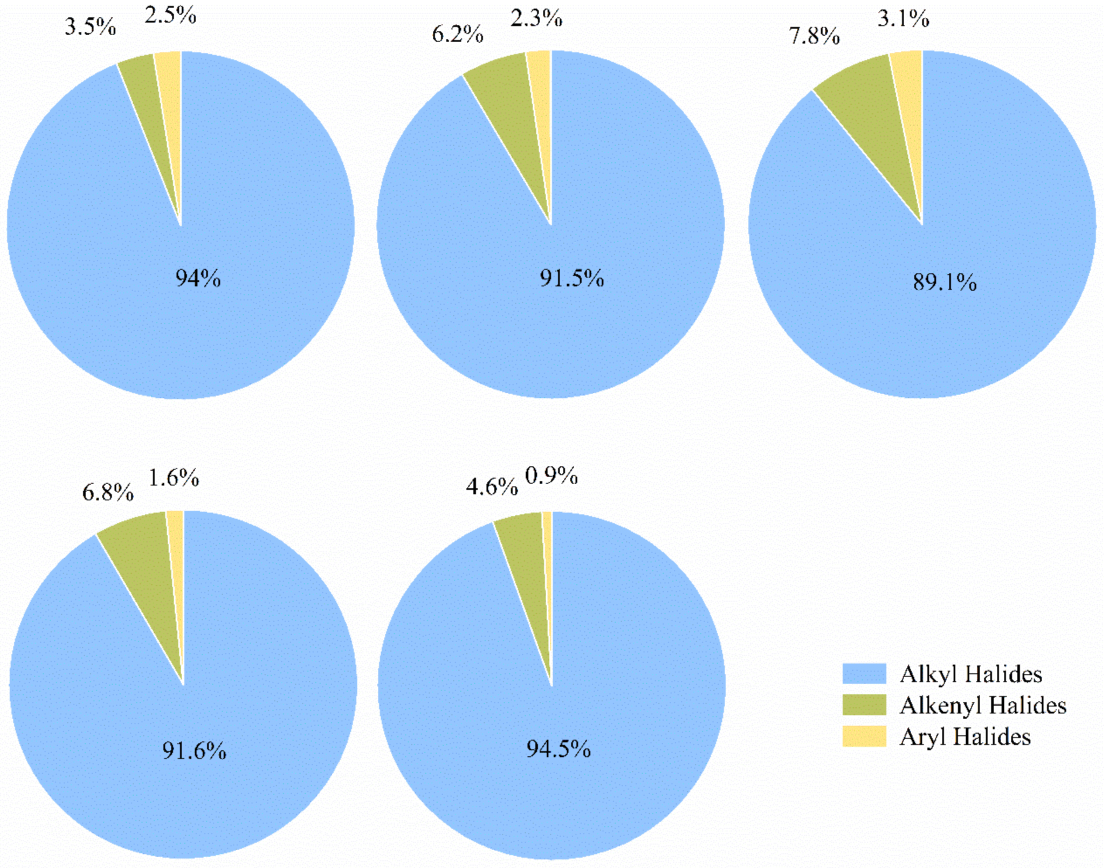

Fig. S1 Proportion of halocarbon chemical components from 2018 to 2022.

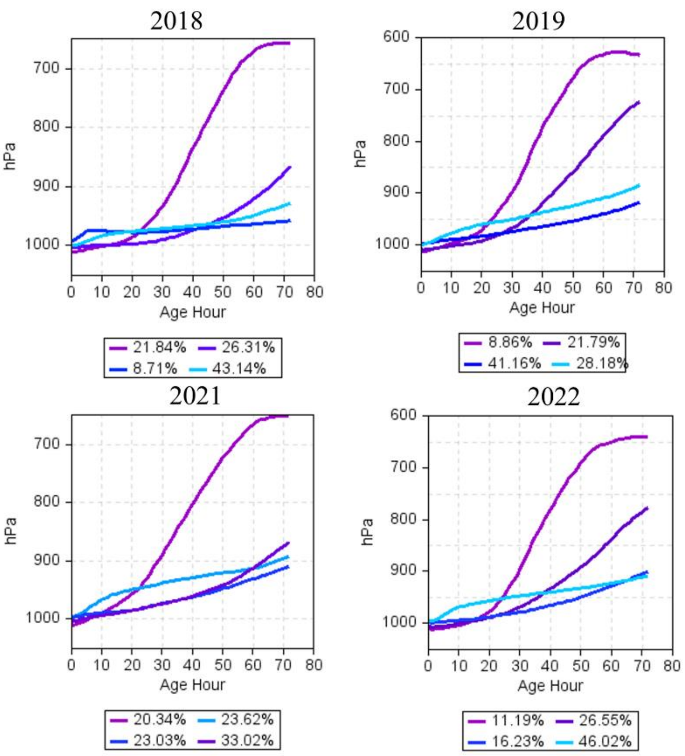

Fig. S2 The height and contribution of each source during 2018-2022.
